# Characterizing the Impact of Fabrication Methods on Mechanically Tunable Gelatin Hydrogels for Cardiac Fibrosis Studies

**DOI:** 10.3390/bioengineering12070759

**Published:** 2025-07-13

**Authors:** Jordyn Folh, Phan Linh Dan Tran, Renita E. Horton

**Affiliations:** 1 Cardiovascular Tissue Engineering Laboratory, Biomedical Engineering Department, Cullen College of Engineering, University of Houston, Houston, TX 77204, USA; jmfolh@cougarnet.uh.edu (J.F.); ptran41@cougarnet.uh.edu (P.L.D.T.); 2 William A. Brookshire Department of Chemical and Biomolecular Engineering, Cullen College of Engineering, University of Houston, Houston, TX 77204, USA

**Keywords:** hydrogel, modulus, mechanotransduction, fibroblasts, fibrosis

## Abstract

The mechanical properties of the extracellular matrix critically influence cell behavior in both physiological and pathophysiological states, including cardiac fibrosis. In vitro models have played a critical role in assessing biological mechanisms. In this study, we characterized mechanically tunable enzymatically crosslinked gelatin-microbial transglutaminase (mTG) hydrogels for modeling cardiovascular diseases. Gelatin hydrogels were fabricated via direct mixing or immersion crosslinking methods. Hydrogel formulations were assessed using the Piuma nanoindenter and Instron systems. This study investigates the effects of fabrication methods, UV ozone (UVO) sterilization, crosslinking methods, and incubation media on hydrogel stiffness. Further, this study examined the response of murine cardiac fibroblasts to hydrogel stiffness. The hydrogels exhibited modulus ranges relevant to both healthy and fibrotic cardiac tissues. UVO exposure led to slight decreases in hydrogel modulus, while the fabrication method had a significant impact on the modulus. Hydrogels incubated in phosphate buffered saline (PBS) were stiffer than those incubated in Medium 199 (M199), which correlated with lower pH in PBS. Fibroblasts cultured on stiffer hydrogels display enhanced smooth muscle actin (SMA) expression, suggesting sensitivity to material stiffness. These findings highlight how fabrication parameters influence the modulus of gelatin-mTG hydrogels for cardiac tissue models.

## 1. Introduction

The extracellular matrix (ECM) provides mechanical support for cells and plays a central role in cellular function contributing to the complex, dynamic cellular and tissue microenvironment [1]. Traditional in vitro systems have been instrumental in advancing biomedical knowledge, however these models do not adequately recapitulate features of the extracellular microenvironment [2]. Organs-on-chip (OOC) models aim to incorporate native microenvironmental features to investigate development, diseases, and cell responses to biomolecular stimuli [3,4,5]. Hydrogels remain a promising material for building tissue models. These biocompatible hydrogels can mimic various features of the native ECM, are mechanically tunable, and can also be modified by dynamic cellular processes in situ [6]. Gelatin, a derivative of collagen, has been widely used in standard cell culture to support cellular adhesion and proliferation [7]. Furthermore, gelatin has also been utilized in tissue engineering applications [8,9,10]. However, unmodified gelatin hydrogels are not suitable for tissue engineering applications as they melt at elevated temperatures. This requires the use of crosslinking agents to enhance thermal stability at physiological temperatures. A variety of techniques have been implemented for crosslinking gelatin including glutaraldehyde [11], genipin [12,13,14,15,16], Irgacure [17,18] and ultra-violet light [19,20]. Microbial transglutaminase (mTG), an FDA approved natural enzyme, has been used in numerous applications to crosslink gelatin to create biocompatible tissue scaffolds. Transglutaminase is present throughout mammalian ECM and facilitates biological processes such as cell adhesion, migration, and proliferation, along with ECM remodeling. Chau et al. found that biocompatible collagen scaffolds treated with transglutaminase showed enhanced cell attachment [21]. In addition to enhancing thermal stability, these crosslinkers can also be used to mechanically tune gelatin hydrogels for in vitro applications, including OOC chip development.

Cellular mechanotransduction plays a role in numerous biological processes including embryonic development, wound healing, tissue regeneration, and fibrosis. The substrate stiffness influences cell health and behavior [22,23,24]. Yeung et al. studied the effect of substrate stiffness on aortic endothelial cells and demonstrated abrupt changes in shape, cytoskeleton assembly and cell spreading when cultured on soft or stiff substrates [25]. Recent studies further highlight how substrate stiffness affect cardiomyocyte contractility and transcription profiles [26] and regulates endothelial cell stiffness through actin cytoskeleton remodeling [27]. These studies show that substrate stiffness influences cell function and morphology. Therefore, materials used to fabricate OOC models must be carefully selected as they can influence results. Studies suggest that mechanical cues from cell culture substrates can influence cell morphology, gene and protein expression, and cell function [28,29,30,31,32]. Therefore, it is important to characterize the mechanical properties of cell culture substrates.

In this study, we report on the modulus of mechanically tunable enzymatically crosslinked gelatin hydrogel, investigate the impact of fabrication methods on hydrogel mechanical properties, and show the influence of modulus on cardiac fibroblasts. Gelatin hydrogel formulations were tested to evaluate the mechanical properties and assess suitability for cardiovascular-related studies. Initial studies suggested that mTG concentrations ranging from 0.8% to 1% were suitable to create hydrogels within the mechanical range of healthy and diseased heart tissue. Hence, this study will characterize the behavior of the hydrogels resulting from these formulations when exposed to cell culture environments. We first assessed the modulus of gelatin thin film chips and gelatin hydrogel cubes. Further, we examine the impact of UV ozone (UVO) exposure on hydrogel mechanical properties. We then evaluated the impact of the modulus change on cardiac fibroblasts through cell morphology, mechanical properties, and protein expression assessments. We assert that this system is suitable for modeling cardiac diseases. Specifically, we advance the application of gelatin-mTG hydrogels by analyzing the effects of key fabrication parameters on mechanical stability and modulus over time. These findings are critical for the reproducibility and reliability of in-vitro cardiac models. This work supports the use of tunable hydrogels in organs- on-chip models for cardiac disease studies by: (1) establishing a tunable platform that mimics the mechanical properties of healthy and fibrotic cardiac tissue, (2) providing insight into how crosslinking methods affect hydrogel mechanics, (3) comparing measurement methods, and (4) providing evidence of cellular response to modulus changes.

## 2. Materials and Methods

### 2.1. Polydimethylsiloxane (PDMS) Stamp Preparation

PDMS stamps were prepared using Sylgard 184 silicone elastomer kit (Dow Corning, Midland, MI, USA) at a 10:1 ratio, base to curing agent. The mixture was poured into a 60 mm petri dish, degassed, and cured overnight at 65 °C. The 10 mm × 10 mm blocks were cut from the cured PDMS elastomer to serve as the stamps to create a flat gelatin surface for the gelatin thin films described below.

### 2.2. Glass Preparation for Gelatin Thin Film Chips

Cover glass (10 mm × 10 mm) was masked using low-adhesive tape (Patco, Evansville, IN, USA). Windows were cut into tape using a laser engraver (Epilog, Golden, CO, USA) to expose the center portion of the glass which allowed for control of the hydrogel thickness, approximately 112 µm. Cover glass was silanized to enhance gelatin adhesion by first preparing it with sodium hydroxide and ethanol, treating with (3-Aminopropyl) triethoxysilane (APTES, Thermo Fisher Scientific, Waltham, MA, USA), rinsing with water, and exposing to glutaraldehyde (Electron Microscopy, Hatfield, PA, USA) for 30 min The glass was rinsed with water and allowed to dry before casting gelatin films.

### 2.3. Gelatin Hydrogel Preparation

This study seeks to assess the suitability of gelatin-mTG hydrogels for modeling cardiovascular diseases. We first identified an appropriate mTG concentration to yield moduli comparable to diseased and healthy heart tissue [33]. Table 1 summarizes the characterized hydrogel formulations, and the experimental parameters examined in this study.

#### 2.3.1. Direct Mix Gelatin Crosslinking

**Figure 1:**
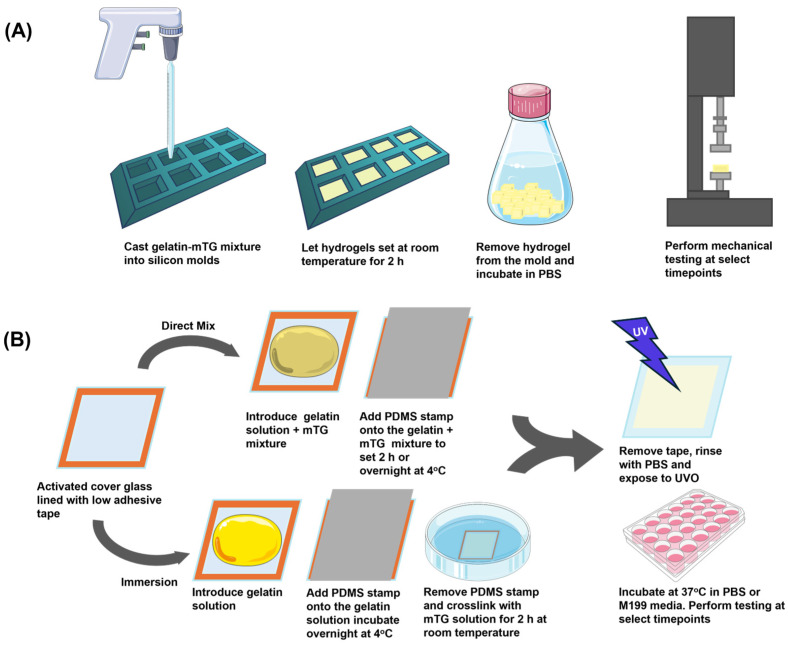
Hydrogel preparation. Gelatin hydrogels were prepared in two formats: cubes for Instron testing and thin films for Piuma nanoindentation and cell culture. (**A**) Gelatin hydrogel cubes were prepared by casting sterile gelatin-mTG mixture into silicon molds. The hydrogels were allowed to set at room temperature before being removed from the mold and the resulting hydrogel cubes were incubated in PBS at 37 °C until the mechanical testing was performed. (**B**) Gelatin thin films were prepared by the direct mix and immersion method. For direct mix: A gelatin- mTG mixture was prepared and cast onto a glass slide. A polydimethylsiloxane (PDMS) stamp was used to create a sandwich, thus controlling the thickness of the thin film. The stamp was removed, and films were incubated in PBS or M199 media. For immersion crosslinking: Gelatin solution was cast onto a glass slide, then sandwiched using a PDMS stamp and allowed to set. The stamp was removed, and the thin films were crosslinked via immersion in an mTG bath, followed by incubation in PBS or M199 media.

Ethylene oxide treated gelatin powder (300 bloom Type A, Sigma-Aldrich, St. Louis, MO, USA) was dissolved in PBS (Cytiva, Marlborough, MA, USA) at 55 °C until the powder was fully dissolved resulting in 4%, 8%, 10%, and 20% solutions. Similarly, mTG-TI powder (Moo Gloo, Modernist Pantry, Eliot, ME, USA) was dissolved in PBS at room temperature creating 1.6% and 2% solutions. The mTG solution was filtered using a 0.22 µm filter prior to adding to the gelatin for crosslinking. To create gelatin hydrogels, equal volumes of gelatin and mTG solutions were mixed yielding final concentrations of 4% or 5% gelatin hydrogels with 0.8% mTG, or 2% or 10% gelatin hydrogels with 1% mTG. This is equivalent to 11 U/mL of gelatin (0.8% mTG) and 13.7 U/mL of gelatin (1% mTG). The hydrogel solutions were cast into 30 mm × 30 mm sterile square silicon molds and allowed to set at room temperature for 2 h before removing the gelatin hydrogel cubes from the molds (Figure 1A). The 0-h samples were immersed in PBS and measured using the Instron system. Samples were incubated at 37 °C in PBS or Medium 199 (M199) (Gibco, Waltham, MA, USA) serum free media to simulate a cell culture environment and measured at 24 h, 48 h, and 72 h

For the gelatin thin film chip preparation, a gelatin-mTG mixture was added to the exposed portion of the 10 mm × 10 mm cover glass ensuring no bubbles were present. The PDMS stamp was then placed onto the gelatin solution to ensure a flat surface for the gelatin films (Figure 1B). The chips were allowed to set at room temperature for 30 min, followed by a 2-h incubation for 5% and 10% gelatin hydrogel and a 24-h incubation for 2% and 4% gelatin hydrogel at 4 °C. The extended incubation time for the lower concentration hydrogels was due to the initial softness of the resulting hydrogel. After 2 h of crosslinking, the 2% and 4% gelatin films remained too fragile for reliable detection by the nanoindenter system. Thus, a 24-h setting period was used to ensure adequate stabilization of the hydrogel surface prior to measurements. The PDMS stamp was then removed, and the gelatin thin film was sterilized by UVO exposure for 3 min. The chips were incubated at 37 °C in PBS or M199 serum free media to simulate a cell culture environment for up to 72 h The thickness of the gelatin layer on the prepared chips were approximately 112 µm and was controlled by the thickness of the adhesive tape used to line the cover glass. Therefore, the thickness of gelatin hydrogel should be sufficiently thick to minimize the effects of an underlying rigid cover glass during mechanical assessments.

#### 2.3.2. Immersion Gelatin Crosslinking

**Figure A1:**
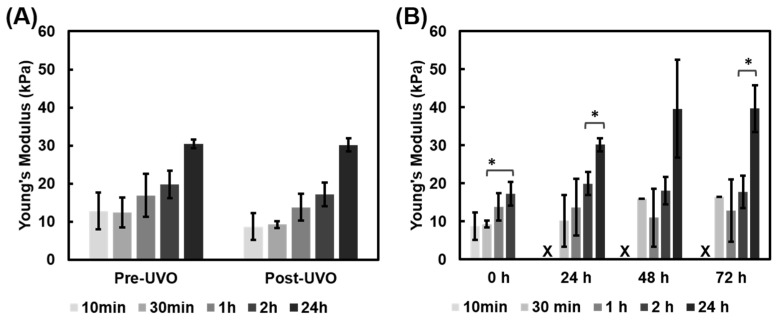
Comparison of mTG incubation for gelatin hydrogel crosslinking. (**A**) Gelatin thin films were exposed to UVO to determine the impact on mechanical modulus. (**B**) Assess mechanical stability over time. 5% gelatin hydrogel films were incubated for 10 min, 30 min, 1 h, 2 h, and 24 h to determine an adequate crosslinking time to generate stable gelatin hydrogels. Increasing the mTG incubation time resulted in a higher hydrogel modulus. Hydrogels exposed to mTG for 30 min or less showed less thermal stability as four of the six samples dissolved prior to the completion of the study. Incubating the hydrogel thin films for 2 h yielded thermally stable hydrogels with moduli within the range of healthy heart tissue. “X” means the samples dissolved before the measurement was collected. Significance was determined via student’s *t*-test (* *p* < 0.05). Data represent SD (*n* = 3, with 2–3 replicates).

Gelatin powder was dissolved in PBS at 55 °C until the powder was fully dissolved, creating 2%, 4%, 5%, and 10% solutions. The gelatin solutions were cast onto activated glass lined with low adhesive tape and sandwiched using a PDMS stamp to control gelatin thin film thickness. The samples were allowed to set at room temperature for 30 min followed by an overnight incubation at 4 °C. The PDMS stamps were removed, and the thin film chips were then immersed in an mTG (0.8% solution for 4% and 5% gelatin hydrogels and 1% solution for 2% and 10% gelatin hydrogels) bath for 2 h at room temperature on a rocker to crosslink the hydrogel (Figure 1B). A 2-h incubation was determined to be the optimal for crosslinking and enhancing thermal stability while maintaining a modulus comparable to direct-mix gelatin thin films (Figure A1). The gelatin thin film chips were subsequently rinsed with PBS and incubated at 37 °C in PBS or M199 serum free media to simulate a cell culture environment for up to 72 h for mechanical testing. Pre-UVO measurements were taken after 2 h of crosslinking and post-UVO measurements were taken immediately after exposure to UVO.

### 2.4. Piuma Nanonindentation

**Figure A2:**
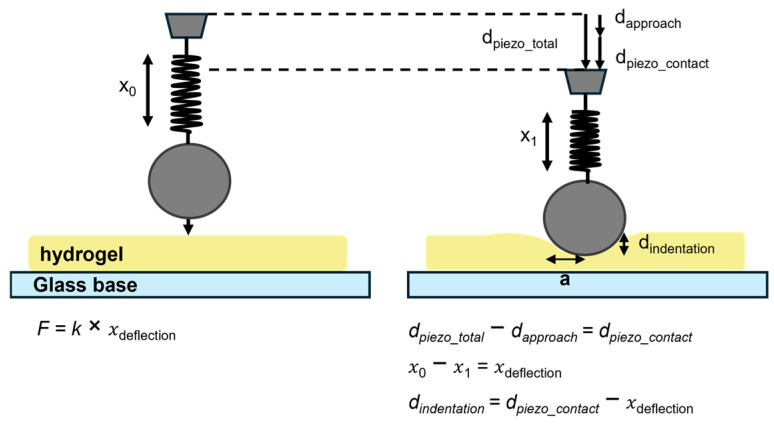
Diagram of nanoindentation probes used for determining modulus. The nanoindentation device uses a spherical probe on a spring attached to a cantilever to record deformation of the sample through measuring the deflection of the cantilever. The compression of the spring as the probe indents into a sample is recorded (*x*) and combined with a known spring constant (*k*), the force can be determined via Hooke’s Law, F = k × *x*. The distance of indentation is calculated by subtracting the total distance of the piezo contact by the spring deflection. The tip radius of the probe and geometric factor are used to calculate the Young’s modulus using the Hertz equation of a sphere and half-space (Equation (1)).

For characterization of sterilization on the gelatin thin film chip modulus, a set of samples were immediately assessed after crosslinking, then assessed again after 3 min of UVO exposure. The remaining samples were sterilized, then rinsed three times with PBS and placed in an incubator. Samples were then assessed at 24 h, 48 h, or 72 h in PBS or serum-free M199 media using the Piuma nanoindenter (Optics11Life, Amsterdam, The Netherlands). The Piuma uses a spherical probe which depresses into a sample. The sample deformation is detected by measuring the deflection of the cantilever (Figure A2). Probes were selected using the Optics11 Life calculator to determine a suitable stiffness relative to the sample modulus. Probes with a stiffness range of 0.48–0.51 N/m, a tip radius 49–50.5 µm, and a geometric factor of 2.92–3.22 (in air) were used for the 2% and 4% gelatin hydrogels. Probes with a stiffness range of 3.06–3.58 N/m, a 48–50 µm tip radius, and a geometric factor of 2.86–3.45 (in air) were used for the 5% and 10% gelatin hydrogels.

A 5 × 5 point matrix scan, for a total of 25 points with regions 50 µm apart, was performed to collect indentation data. Mechanical stiffness calculations were performed using the Peak Load Poke method with a maximum load of 1–30 µN and a piezo speed of 3 µm/s. Load-displacement curves were generated using the Piuma Nanoindenter Software (v3.5.0). Optics11 DataViewer Software (v2.7.0) was used to calculate the modulus from the load-displacement curves. All calculations utilize the Hertzian contact model, with indentation depth of no more than 16% of the probe tip radius. An R^2^ value of 0.98 was used as the cutoff for acceptable model fitting.

The probe spring constant measured spring deflection, and the probe displacement into the sample are used to calculate the load force (Equation (1)). The Effective Young’s modulus can be calculated using the tip radius and the formula for Hertzian contact between a sphere and an elastic half-space, where the half-space is defined as an infinite, elastic plane. Because the size of the gelatin thin film is orders of magnitudes larger than the probe, the thin film can be simplified as a half-space and the following equation can be applied [34]:

(1) $$F\;=kx=\;\frac{4}{3}E_{eff}\sqrt{R_{i}}\cdot h^{\frac{3}{2}},$$

where,

*F* = load force

*k* = spring constant

*x* = spring deflection

*E_eff_* = Effective or Reduced Young’s Modulus

*R_i_* = probe radius

*h* = displacement

The Young’s Modulus can then be calculated using the relationship between Young’s Modulus and Effective Young’s Modulus using Poisson’s Ratio (Equation (2)):

(2) $$E\;=E_{eff}\left(1-v^{2}\right)$$

where,

*E* = Young’s Modulus

*E_eff_* = Effective or Reduced Young’s Modulus

*v* = Poisson’s Ratio, where the ratio for a fully hydrated hydrogel is 0.5 [35]

### 2.5. Instron Mechanical Testing

The dimensions of the gelatin cubes, width and thickness were measured using a digital caliper (Model No. CD-6” CS, Mitutoyo, Aurora, IL, USA). The mechanical modulus of the gelatin cubes was measured using a static load cell with the 5943 Instron mechanical tester (Instron, Norwood, MA, USA). Samples were subjected to a compressive strain of 30 N at a constant extension rate of 0.20 mm/s. The control mode was set to a preload extension of 5 mm/mm, applying a preload force of 5 mN. The strain response under the applied compressive load was recorded using Blue Hill 3.31 software (Instron, Norwood, MA, USA). Stress-strain curves were generated, and the slope of the linear region was calculated to determine the Young’s modulus of the hydrogel samples. Young’s modulus was calculated using the formula from Hooke’s Law (Equation (3)).

*E* = *σ*/*ε* = (*F_n_*/*A*)/(*dl*/*l_o_*) (3)

where,

*E* = Young’s modulus (N/m^2^)

*ε* = strain

*σ* = normal stress

*F_n_* = normal force acting perpendicular to the area

*A* = area

*dl* = change of length

*l_o_* = initial length

### 2.6. Cardiac Fibroblast Culture

All animal protocols were approved by the Institutional Animal Care and Use Committee at the University of Houston. A modified version of a previously published harvest protocol was used in this study [36]. Briefly, neonatal C57BL/6 mouse cardiac fibroblasts were obtained by digesting the hearts of two-day old mice in 1 mg/mL trypsin (Affymetrix, Santa Clara, CA, USA) solution for 4 h at 4 °C. The trypsin reaction was stopped by adding warm media containing 10% fetal bovine serum (FBS, Gibco, Waltham, MA, USA). The tissue was transferred to a collagenase solution and mechanically dissociated (Worthington Biochemical, Lakewood, NJ, USA). The cell suspension was centrifugated at 1200 rpm for 10 min at 4 °C. Cells were resuspended in Hank’s Balanced Salt Solution, passed through a 40 µm strainer, and centrifuged at 1200 rpm for 10 min at 4 °C. The cells were then pre-plated to separate cardiomyocytes and fibroblasts. Fibroblasts were allowed to attach to the flask and were cultured in M199 basal media supplemented with 2 mM L-glutamine, 0.1 mM nonessential amino acids, 10 μM HEPES, 19.4 μM glucose (Sigma-Aldrich, St. Louis, MO, USA), 50 U/mL of penicillin, 1.5 μM B12 (Sigma-Aldrich, St. Louis, MO, USA) and 10% FBS until confluent. Fibroblasts were seeded onto the surface of the hydrogel chips at approximately 14,700 cells/cm^2^. Cells were fixed and stained at 72 h post seeding.

### 2.7. Immunocytochemistry and Analysis

Primary mouse cardiac fibroblasts were fixed and permeabilized for 15 min at room temperature with 4% paraformaldehyde (Electron Microscopy, Hatfield, PA, USA) and rinsed three times with PBS. The cells were blocked with 1% bovine serum albumin (BSA, GoldBio, Olivette, MO, USA). Fibroblasts were incubated with primary antibodies against vimentin (1:200, Novus Biologicals, Centennial, CO, USA) and smooth muscle actin (1:800, Proteintech, Rosemont, IL, USA) overnight at 4 °C. The samples were then rinsed three times with PBS and incubated with phalloidin conjugated with Alexa Fluor 633 (Invitrogen, Carlsbad, CA, USA), 4′,6-diamidino-2-phenylindole (DAPI) (Thermo Fisher Scientific, Waltham, MA, USA) goat anti-rabbit 488 (Invitrogen, Carlsbad, CA, USA), goat anti-chicken 546 (Invitrogen, Carlsbad, CA, USA) for one hour. Cells were rinsed three times with PBS and imaged using the Leica SP8 confocal microscope (Leica, Wetzlar, Germany). Immunofluorescence quantification for SMA was conducted using Fiji ImageJ x64 [37]. We first created a Sum Z stack projection image. The background was subtracted from the signal and the average intensity was calculated from minimum of 8 regions of interests per image with at least 5 images per sample. The cell nucleus was stained with DAPI and captured through Z-stack imaging. Image processing was performed using Fiji, where Z-stacks were merged and smoothed to enhance nuclear definition. The threshold feature was applied to isolate nuclei through positive DAPI staining, followed by the “analyze particles” function to calculate nuclear area and nuclear aspect ratio.

### 2.8. Statistical Analysis

Statistical assessment of modulus changes, effect of incubation media on the modulus of the gelatin-mTG hydrogels, hydrogel cube dimensions, and pH of the incubation solution, were conducted using student’s *t*-test. Significance was determined using *p*-values less than or equal to 0.05.

## 3. Results

### 3.1. Elastic Modulus Studies

#### 3.1.1. UVO Impact on Gelatin Film Modulus

**Figure 2:**
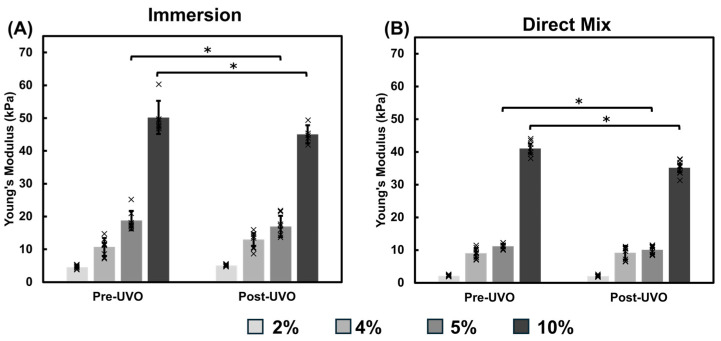
UVO Impact on Hydrogel Modulus. The Young’s modulus of gelatin-mTG hydrogels were assessed before and after UVO exposure. (**A**) Mechanical assessments reveal that hydrogels crosslinked via the immersion method show slight decreases in the modulus of the 5% and 10% hydrogels. (**B**) Hydrogels crosslinked via the direct mix method also show decreases in the modulus of the 5% and 10% hydrogels as a result of UVO exposure. Significance was determined using student’s *t*-test (* *p* < 0.05). Data represent mean ± SD (*n* = 3, with 3 repeats each). Individual data points are represented by x.

The crosslinked gelatin thin films were sterilized by UVO exposure prior to culturing cells or beginning the incubation study. We found that the temperature within the UVO system rises at a rate of approximately 3.7 °C/min. After three minutes, the temperature within the chamber rose from 20 °C to 31 °C. Thus, we investigated whether UVO exposure would impact the modulus of gelatin films. Gelatin films crosslinked via the immersion and direct mix method were tested before and after UVO exposure (Figure 2). The lower concentration gelatin hydrogels, 2% and 4%, crosslinked via the immersion method exhibited minimal differences in response to UVO exposure (Figure 2A). However, the 5% and 10% gelatin thin films exhibited a slight decrease in modulus after UVO exposure but remained within physiological or pathophysiological stiffness ranges, respectively (Figure 2A). Similar to the immersion crosslinked hydrogels, the 2% and 4% direct mix hydrogels also showed minimal impact from UVO exposure. The 10% hydrogels crosslinked via direct mix showed a decrease in modulus from 41 kPa to 35 kPa as a result of UVO exposure (Figure 2B). This suggests that UVO exposure led to modest softening within the stiffer hydrogels.

#### 3.1.2. Mechanical Properties of Gelatin Film Chips Using Piuma

Gelatin-mTG hydrogels crosslinked using the direct mix or immersion methods were measured with the Piuma nanoindenter to assess the mechanical modulus. The prepared chips were incubated in cell culture conditions in PBS or M199 at 37 °C and 5% CO_2_ for the duration of the study. Gelatin thin films were assessed at 0 h, 24 h, 48 h, and 72 h timepoints. The following parameters were assessed: gelatin concentration, crosslinking method, and immersion solution to determine the impact on gelatin thin film modulus.

##### Mechanical Properties of Gelatin Films Using the Immersion Crosslinking Method

The effect of mTG crosslinking times on hydrogel modulus and thermal stability was assessed by crosslinking 5% gelatin thin films in a 0.8% mTG bath for 10 min, 30 min, 1 h, 2 h, and 24 h at room temperature. The moduli of the thin films were assessed after UVO sterilization and over a 72-h period of incubation in PBS at 37 °C (Figure A2).

Gelatin thin films crosslinked in a mTG bath for 10 min and 30 min were softer than hydrogels that were crosslinked for at least 2 h suggesting incomplete crosslinking (Figure A2). Furthermore, all 10 min samples and the majority of the 30 min samples dissolved after incubation at 37 °C. There was no significant change in modulus for samples incubated in mTG for 1 or 2 h at any timepoints; however, the 2-h incubation samples were more stable throughout the 72-h relative to the 1-h samples (Figure A2). The 24-h immersion hydrogels had a significantly higher modulus than the 2-h immersion hydrogels, and by 72 h remained significantly higher than the 2-h hydrogels at approximately 40 kPa. The 2-h hydrogel samples exhibited thermal stability and were within a suitable modulus range for heart model applications at 18 kPa. Therefore, a 2 h mTG crosslinking time was selected for the remaining immersion-based studies.

**Figure 3:**
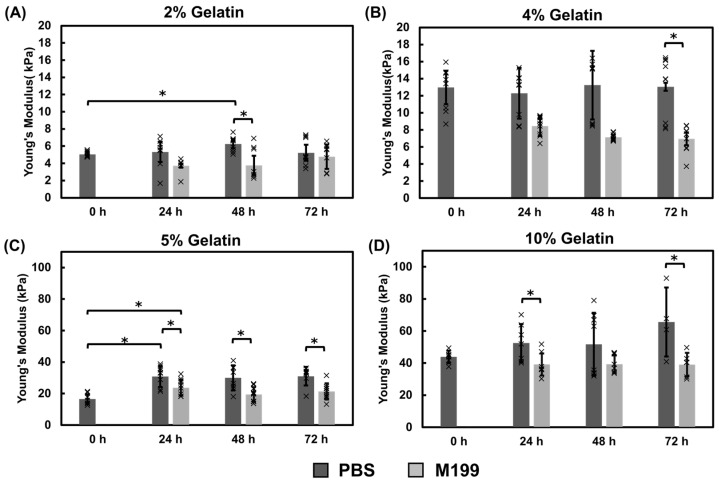
Mechanical assessment of immersion-crosslinked gelatin thin film chips. The modulus of (**A**) 2%, (**B**) 4%, (**C**) 5%, and (**D**) 10% gelatin thin films were assessed after incubation at 37 °C in PBS or M199 media at 24 h, 48 h, and 72 h Hydrogels incubated in PBS were stiffer than those incubated in M199. Significance was determined using student’s *t*-test (* *p* < 0.05). Data represent the mean ± SD with *n* = 3–5. Individual data points are represented by x.

Gelatin thin films were crosslinked by placing the gelatin chips into a 0.8% mTG or 1% mTG bath for 2 h Following UVO exposure, hydrogel moduli were measured over a 72-h period, with the post-UVO measurements serving as the baseline (0-h) comparison (Figure 3). The modulus of the 2% hydrogels did not significantly increase over the three-day period, with an approximate modulus of 5 kPa, independent of incubation media (Figure 3A). The 4% hydrogels incubated in PBS had a modulus of approximately 13 kPa, however by 72 h, hydrogels incubated in M199 media were significantly softer and were found to be around 7 kPa (Figure 3B). The 5% hydrogels exhibited a significant increase in modulus for both incubation solutions after 24 h at 37 °C, increasing from approximately 16 kPa to 30 kPa in PBS and 23 kPa in M199 (Figure 3C). After an initial stiffness increase in the first 24 h, the hydrogels did not exhibit any significant changes in modulus. Furthermore, hydrogels incubated in PBS were significantly stiffer than hydrogels incubated in M199 media (Figure 3C). The 10% hydrogels incubated in PBS had significantly higher modulus measurements relative to hydrogels incubated in M199 media at 72 h with an average modulus of 65 kPa in PBS and 39 kPa in M199 (Figure 3D). These data suggest that the incubation time and incubation solution can influence the modulus of gelatin hydrogels.

##### Mechanical Properties of Gelatin Films Using the Direct Mix Crosslinking Method

**Figure 4:**
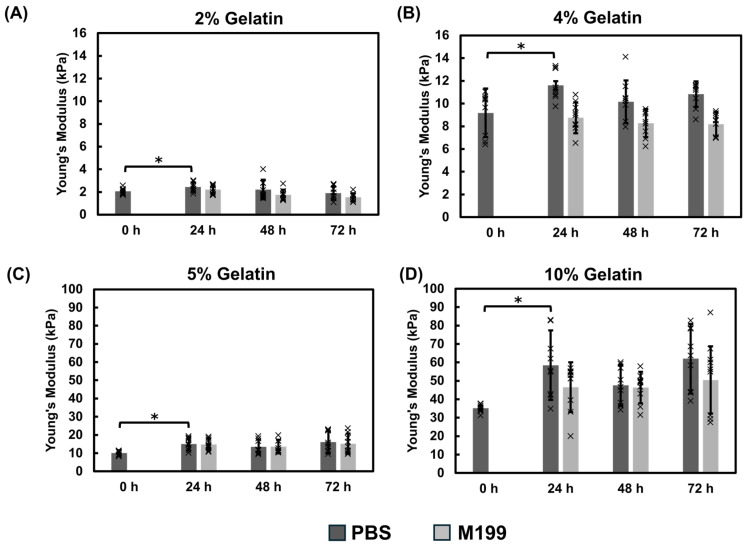
Mechanical assessment of direct mix crosslinked thin films. (**A**) 2%, (**B**) 4%, (**C**) 5%, and (**D**) 10% gelatin thin films were incubated in PBS or M199 media to determine the effects of incubation solutions on the mechanical modulus. Significance was determined via student’s *t*-test (* *p* < 0.05). Data represent the mean ± SD (*n* = 3, 3 replicates). Individual data points are represented by x.

The direct mix gelatin thin films showed similar behavior to the immersion crosslinking with an initial increase in modulus during the first 24 h of incubation. The thin films incubated in PBS exhibited a higher modulus relative to those incubated in M199 culture medium, particularly 4% and 10% gelatin concentrations. For the 2% gelatin films, the modulus averaged approximately 2 kPa in both PBS and M199 over the 72-h period (Figure 4A). The 4% gelatin thin films exhibited a modest difference between the two incubation solutions, averaging 10 kPa in PBS and 8 kPa in M199 (Figure 4B). The 5% gelatin thin films showed similar stiffnesses, approximately 15 kPa, independent of the media (Figure 4C). Lastly, 10% gelatin samples maintained consistent mechanical properties, with PBS samples measuring around 51 kPa and M199 samples averaging 48 kPa after 72 h (Figure 4D). While the M199 samples showed a slightly lower modulus, the difference was not statistically significant.

#### 3.1.3. Comparison of Fabrication Methods and pH Effects

**Figure 5:**
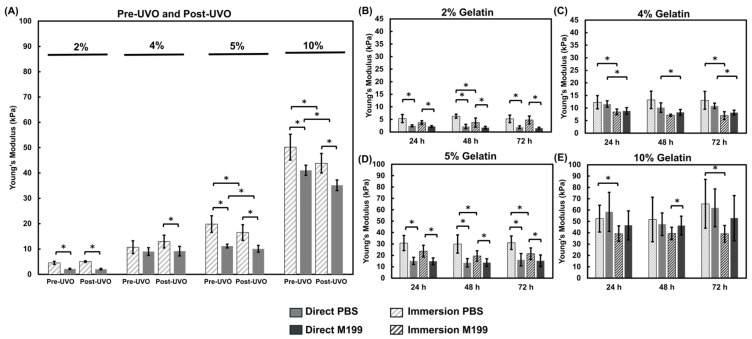
Comparison of Young’s Modulus measurements for gelatin thin film chips based on crosslinking methods and incubation solution. The immersion (pattern) and direct mix (solid) crosslinking methods and the incubation solution were assessed to determine the effects on the thin film chips modulus. (**A**) The moduli of the thin film chips were compared before (Pre-UVO) and after UVO (Post-UVO) exposure. These studies were conducted in PBS only as these measurements were conducted prior to starting the 37 °C incubation studies. (**B**–**E**) Crosslinking methods were compared with respect to their incubation solution for (**B**) 2%, (**C**) 4%, (**D**) 5%, (**E**) 10% gelatin hydrogels. Significance was determined via student’s *t*-test (* *p* < 0.05). Data represent the mean ± SD (*n* = 3, 3 replicates).

Figure 5 provides an overview of the effects of crosslinking methods, incubation solutions, and UVO exposure on hydrogel modulus. We first examined the impact of the crosslinking method and UVO exposure on the modulus prior to incubating the thin film chips (Figure 5A). Results showed that the thin films fabricated using the immersion crosslinking method were significantly stiffer than the direct mix thin films before and after UVO exposure (Figure 5A). Additionally, the 5% and 10% thin films showed a modest decrease in modulus after UVO exposure in both the direct and immersion crosslinking methods. The UVO effects were minimal in the 2% and 4% thin films (Figure 5A). The immersion crosslinked 2% and 5% hydrogels were significantly stiffer than the direct mix counterparts for all time points (Figure 5B, D). For the 4% hydrogels, incubation solution appeared to have a greater effect on modulus relative to crosslinking method, with PBS incubation yielding higher modulus values independent of the crosslinking method (Figure 5C). The 10% thin films showed minimal differences when comparing crosslinking methods after 72 h of incubation (Figure 5E). Further, these data suggest that the incubation solution can affect hydrogel stiffness. The immersion crosslinking method showed that PBS incubation resulted in stiffer hydrogels relative to M199 (Figure 5B–D).

We questioned whether pH may be a factor in the observed differences among the PBS and M199 incubations. PBS and M199 solutions were collected and measured over the duration of the experiment for the 5% gelatin crosslinked with 0.8% mTG thin films incubated at 37 °C in a CO_2_ incubator. This formulation was selected for cell studies and therefore was also selected to conduct the pH assessments. Measurements showed that PBS decreased from pH 6.9–7.0 to pH 6.5 by 72 h The M199 media remained stable with a pH 6.9–7.0 (Figure A4). The decrease observed in the PBS may contribute to modulus differences observed in the gelatin hydrogels incubated in PBS.

#### 3.1.4. Mechanical Properties of Gelatin Hydrogel Cubes Using Instron

**Figure 6:**
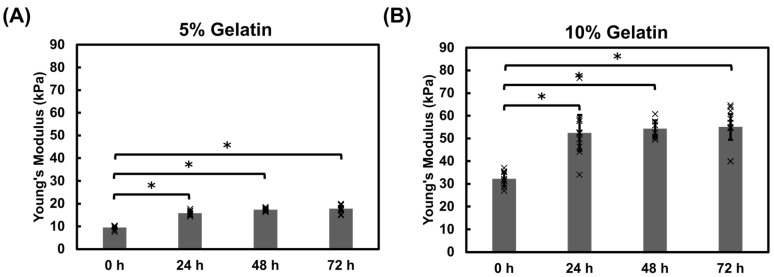
Instron compression testing of direct mix gelatin-mTG hydrogels. (**A**) The 5% and (**B**) 10% gelatin hydrogels exhibited an increase in Young’s modulus during the initial 24 h Significance was determined via a student’s *t*-test (* *p* < 0.05). Data represent the mean ± SD (*n* = 3, with 3 replicates). Individual data points are represented by x.

The immersion method relies on diffusion to facilitate crosslinking, making it an unsuitable method for thick gelatin hydrogels due to limited penetration which lead to insufficient crosslinking. The modulus of the 2% and 4% gelatin hydrogels was only assessed by Piuma as the hydrogels were too fragile to maintain a consistent shape which presented a challenge for obtaining reliable measurements with the Instron system. To assess the bulk properties of the gelatin hydrogels, 5% and 10% gelatin hydrogel cubes were prepared by directly mixing the gelatin and mTG solutions. Both 5% and 10% hydrogels showed an increase in modulus within the first 24 h (Figure 6). The 5% gelatin hydrogels increased from 10 kPa to 16 kPa and the 10% gelatin hydrogels increased from 30 kPa to 53 kPa. This increase in stiffness was also observed in gelatin thin films. This gradual stiffening may be attributed to the continual activity of the microbial transglutaminase and may be useful for investigation of tissue remodeling in diseases where tissue stiffness increases.

**Figure 7:**
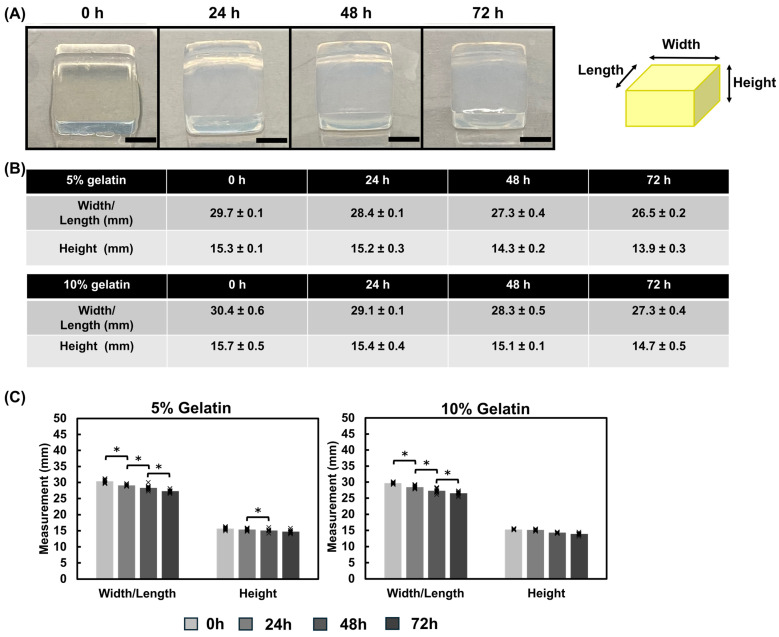
Dimensional assessment of direct mix crosslinked hydrogel cubes. (**A**) Representative images of 5% gelatin hydrogel cubes at 0, 24, 48, and 72 h Scale bar = 1 cm. A schematic diagram illustrates the measured dimensions: width, length, and height. (**B**,**C**) Hydrogel cube dimensions were recorded for the 5% and 10% gelatin hydrogels at each time point prior to performing the Instron measurements. Hydrogels decreased in width/length over the duration of the study. Individual data points are represented by x. Significance was determined via a student’s *t*-test (* *p* < 0.05). Data represents the mean ± SD (*n* = 3, 3 repeats).

Dimensional assessments of the hydrogel cubes revealed gradual contraction over the duration of the study (Figure 7A,B). The width and length measurements decreased by 15–19% of the initial size during the incubation for both hydrogel formulations (Figure 7B,C).

**Figure A3:**
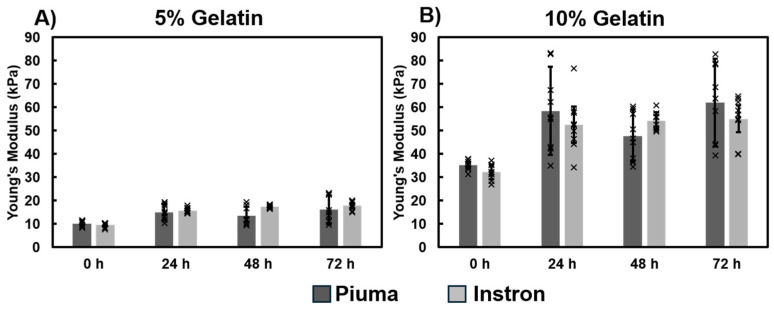
Comparison of Young’s Modulus measurements between Instron and Piuma methods. (**A**) 5% gelatin hydrogels and (**B**) 10% gelatin hydrogels, showed no statistically significant differences between Piuma and Instron mechanical testing methods among the gelatin-mTG formulations. However, the Piuma nanoindentation system exhibited greater variability within measurements likely due to the higher sensitivity of the system relative to the Instron. Data represent mean ± SD (*n* = 3, with 3 replicates). Individual data points are represented by x.

Comparing modulus measurements from the Piuma and Instron systems revealed comparable results (Figure A3). Hydrogels exhibit an initial increase in Young’s modulus within the first 24 h post-fabrication, followed by a plateau for 5% (Figure A3A) and 10% hydrogels (Figure A3B) suggesting continued mTG activity.

### 3.2. Assessing the Mechanical Impact of Modulus Cardiac Fibroblasts

**Figure 8:**
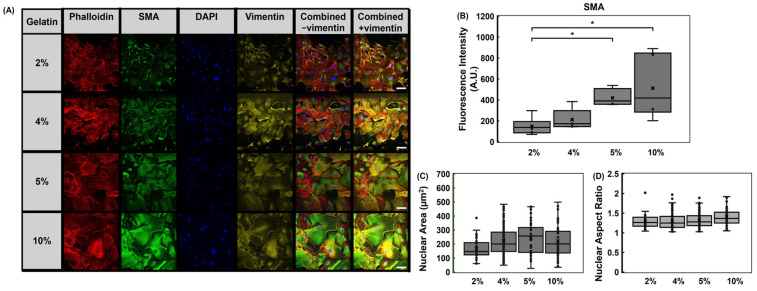
Cardiac fibroblasts cultured for 72 h on 2%, 4%, 5%, or 10% gelatin substrates. (**A**) Cardiac fibroblasts were stained for smooth muscle actin (SMA, green), vimentin (yellow), F-actin with phalloidin (red) and nuclei with DAPI (blue). Vimentin and F-actin expression levels are comparable among the groups. The SMA expression was enhanced with increasing modulus. Scale bar = 100 µm. (**B**) Fluorescence analysis of SMA expression. (**C**) Nuclear area and (**D**) the nuclear aspect ratio was assessed in cardiac fibroblasts. The mean is indicated by “x”. Significance was determined via a student’s *t*-test (* *p* < 0.05). Data represent mean ± SD (*n* = 3).

Fibroblasts play a significant role in cardiac tissue remodeling and in fibrosis. Upon activation, fibroblasts undergo transdifferentiation to myofibroblasts, which are responsible for the matrix deposition in fibrotic tissues [33,38,39]. We questioned whether we could capture morphological changes in fibroblasts in response to hydrogel modulus. We assessed vimentin, SMA, and F-actin expressions within cardiac fibroblasts (Figure 8A). There were no appreciable differences in phalloidin or vimentin expression among the samples. SMA can serve as an indicator for cardiac fibrosis and myofibroblasts [40]. Thus, we examined SMA expression within cardiac fibroblasts cultured on gelatin hydrogels ranging from 2% (2 kPa) to 10% (49 kPa) gelatin concentrations. The lower modulus hydrogels were included to assess whether SMA expression is responsive to changes in mechanical modulus. Results suggest that fibroblasts cultured on 2% gelatin hydrogels of approximately 2 kPa exhibited lower SMA expression relative to fibroblasts cultured on stiffer hydrogels (Figure 8A,B). This observation suggests that SMA expression may be modulated through mechanical cues.

Nuclear morphology, size and aspect ratio, were measured to determine if cell culture substrate modulus can impact nuclear morphology. Nuclear area measurements revealed a slight increase in area with increasing hydrogel modulus between 2% and 5% gelatin hydrogels, although this was not statistically significant (Figure 8C) The nuclear aspect ratio was similar among the hydrogel groups around 1.3 (Figure 8D).

## 4. Discussion

Tissue engineered chip models are powerful tools for investigating cardiovascular disease cascades. Material selection is a critical parameter for chip fabrication because mechanical properties can impact cell function, morphology, and gene and protein expression [28,41,42,43,44,45]. This study aimed to evaluate the modulus of mechanically tunable gelatin-mTG hydrogel formulations to identify mixtures suitable for modeling physiological and pathophysiological cardiac tissues. We selected gelatin-mTG due to its biocompatibility and ease of manipulation. These hydrogels have been used in a variety of studies, including hypertrophy [46], vessel modeling [8,47], and differentiation [48]. However, further mechanical assessments were needed to examine the impact of chip fabrication methods on the hydrogel modulus. This study assesses the effect of gelatin-mTG concentrations, fabrication methods, and UVO sterilization on the hydrogel mechanical modulus. We also show that hydrogel modulus influences protein expression and cardiac fibroblast morphology.

We first assessed the impact of UVO exposure on the hydrogel modulus (Figure 2). The temperature within the UVO chamber increases during exposure which may affect the mechanical modulus of the gelatin-mTG thin film chips. We found that UVO exposure led to slight decreases in the mechanical modulus of the hydrogels relative to the pre-UVO measurements. We observed an 11 °C temperature increase over the exposure period which may have contributed to the observed decrease in modulus. We then examined two methods for crosslinking gelatin hydrogels with mTG: immersion and direct mixing. The immersion hydrogels incubated in PBS yielded the highest modulus values when compared against the direct mix crosslinking (Figure 3, Figure 4 and Figure 5). The immersion method may be suitable for small scale 3D printing or hydrogel patterning which can then be crosslinked as described in this study. The direct mix crosslinking method involves manipulating the hydrogel while it crosslinks, which may result in less consistent designs. Further, the mTG increases the viscosity of the gelatin solution which may present challenges with 3D printing applications. The immersion technique relies on diffusion for crosslinking which poses limits on the thickness of the hydrogels as thicker materials will result in insufficient or partial crosslinking. In contrast, the direct mix method does not pose such limitations. The choice of crosslinking method should be tailored to the application: immersion may suffice for thin films and hydrogel patterning applications, while direct mixing may be preferable for bulk hydrogels or thick (>200 µm) 3D construct applications. It should be noted, the immersion technique uses a higher mTG (w mTG/w gelatin) content, 13.7× relative to the direct mix method to ensure adequate crosslinking since this method relies on diffusion.

**Figure A4:**
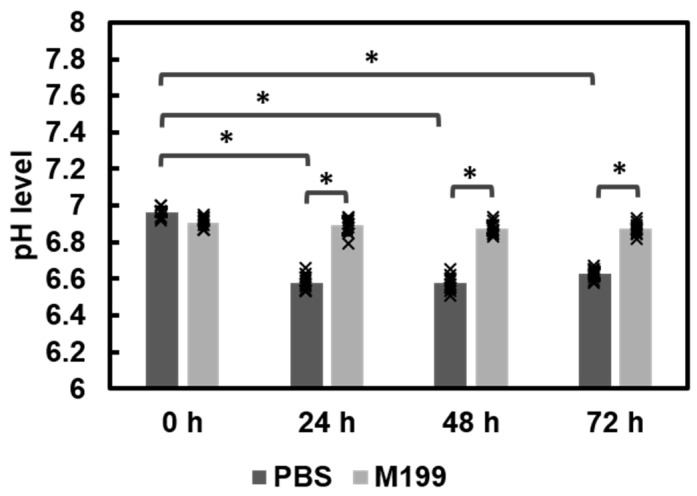
pH measurements of hydrogel incubation solutions. Gelatin-mTG hydrogels were incubated in PBS or serum free M199 media at 37 °C in a CO_2_ incubator for the duration of the study. While both solutions start at a pH of approximately 7, PBS exhibited the greatest fluctuation in pH whereas the M199 media remained relatively stable near pH 7. Significance was determined via a student’s *t*-test (* *p* < 0.05). Data represent mean ± SD (*n* = 3, with 3 replicates). Individual data points are represented by x.

Hydrogels fabricated using the immersion technique exhibited a higher sensitivity to the incubation solution where PBS samples showed higher modulus values relative to the M199 media samples. Previous studies indicate that mTG has optimal activity at pH 6–7 [49]. Our studies revealed that while the M199 media maintained a consistent pH of approximately 7, PBS decreased from 7 to approximately 6.5 (Figure A4). This suggests that the pH level differences of the incubation solution may contribute to the higher stiffnesses observed in the hydrogels incubated in PBS. Hydrogel chips are often stored in buffers such as PBS prior to introducing cells. These results emphasize that storage conditions, testing conditions, and pH monitoring are all critical when characterizing hydrogels. We also show that the gelatin cubes contract over the duration of the experiment (Figure 7), which may contribute to the temporal increase in modulus. Dimensional assessments of the hydrogel cubes revealed up to a 19% decrease in size for the hydrogel cubes. Contraction within the thin film was not appreciably observed likely because the films are immobilized on the glass base whereas the cubes are not constrained or fixed to a surface, thus highlighting the influence of environmental conditions on the mechanical properties of materials. These dimensional changes could impact the long-term stability of hydrogel scaffolds and should be considered in the design of tissue engineering constructs, particularly for applications requiring precise geometries or for 3D tissue models that are not bound to a surface. The gradual stiffening observed within the hydrogels overtime (Figure 3, Figure 4, Figure 5 and Figure 6) could be advantageous for applications requiring time-dependent mechanical adaptation e.g., tissue engineering scaffolds that mimic the evolving stiffness of developing tissues or diseased tissue remodeling. However, this phenomenon can be reduced by inactivating the mTG through exposing the hydrogels to elevated temperatures, approximately 50–60 °C [50], prior to introducing cells.

**Figure A5:**
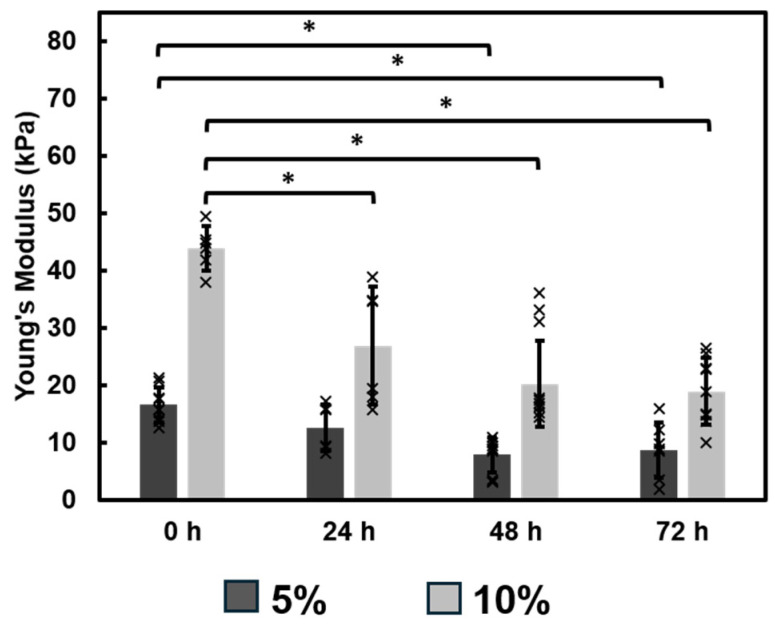
Degradation assessment. Immersion-crosslinked gelatin thin films were incubated in a 1 ng/mL collagenase in M199 media. The collagenase-induced degradation resulted in a decrease in modulus over time. Significance was determined via a student’s *t*-test (* *p* < 0.05). Data represents the mean ± SD (*n* = 3). Individual data points are represented by x.

Table 2 highlights some of the differences between the Piuma nanoindenter and the Instron mechanical tester. In this study, we used compression testing to measure the Young’s modulus of the gelatin hydrogels (aggregate data are included in Table A1). The Piuma nanoindentation system has some similarities with the atomic force microscope in that both systems rely on capturing cantilever deflections to measure mechanical properties. We acknowledge that the systems have different resolution capacities, the Instron system was selected as a comparison due to the system’s versatility, and it is an industry standard. The comparison serves as a validation of the Piuma system for our gelatin chips. We created gelatin thin film chips and cubes which allowed us to assess the gelatin modulus using sample formats that were appropriate for the respective instruments. The measurements between the Instron and Piuma systems showed good agreement among the hydrogels (Figure A3) over the 72-h study. The Piuma system was also suitable for capturing changes in hydrogel surface stiffness after collagenase degradation, highlighting its potential for assessing dynamic surface properties (Figure A5).

Rheological assessments can provide insight into viscoelastic properties such as shear (G′) and loss (G″) moduli. Future studies will focus on the viscoelastic characterization of the hydrogels in a cellularized system. However, this study focused on evaluating Young’s modulus to directly compare with reported heart tissue stiffness values [31]. This study evaluated how fabrication methods affect hydrogel stiffness, providing guidance for developing mechanically tunable cardiac tissue chip models. Although, the elastic modulus of the 4% gelatin-mTG hydrogels more closely matched that of the native heart (8–15 kPa) in comparison to the 5% gelatin-mTG hydrogels, the 4% hydrogels often fractured during chip fabrication processes, limiting their utility as a robust material for chip models. Thus, the 5% gelatin hydrogel was selected to represent the physiological heart modulus, 14–22 kPa, whereas the 10% gelatin hydrogel was used to represent a fibrotic microenvironment, 47–51 kPa, for the fibroblast studies. While this study assessed the modulus of gelatin-mtg hydrogels under static conditions, we acknowledge that native cardiac tissue also experience complex biomechanical stimuli, including cyclic mechanical stretch and fluid shear stress [51]. We present the first steps in assessing the impact of the fabrication process on the mechanical properties of the gelatin-mTG hydrogel. Future studies will focus on a co-culture system incorporating cardiomyocytes and fibroblasts into a gelatin-mTG hydrogel platform which can facilitate assessments of stretch and conduction. Additionally, shear stress can be examined through the incorporation of vascular cells and flow within the chip model. The hydrogels can be patterned to guide cell organization and alignment with the model to mimic the role of matrix in supporting tissue organization. Studies show that substrate stiffness can influence cellular behavior [24,52,53,54]. We show that the mechanical modulus of cell culture substrates can influence protein expression within cardiac fibroblasts. SMA expression is often used to identify myofibroblasts, the cells that deposit extracellular matrix during scar tissue formation [55,56,57]. SMA was found to be in lower abundance in cells cultured on 2% hydrogels (Figure 8). This expression was higher in cells cultured on the 10% hydrogels. Interestingly, SMA expression was also observed in the physiological hydrogels, 4% and 5%. This observation aligns with a study by Herum et.al, which found SMA expression in cardiac fibroblasts cultured on hyaluronic acid hydrogels that mimicked the modulus of the healthy heart [38]. They also observed enhanced SMA expression correlated with increased hydrogel stiffness. This suggests that other critical factors may impact fibroblast activation and the transdifferentiation of fibroblasts to myofibroblasts which may require a combination of assessments to confirm transdifferentiation. We did not observe notable differences in the f-actin or vimentin expression within the fibroblast study (Figure 8). The future goal is to develop a simplified tissue model to investigate fibrosis, therefore, the fibroblast study focused on high cell densities. This presented a challenge in reporting cell spreading or area as we could not adequately measure single cells due to the high cell density. Future studies will utilize gelatin hydrogels to develop cardiac fibrosis models to investigate mechanisms that contribute to heart remodeling, which will include cell viability, cell morphology, proliferation, and gene and protein assessments to probe mechanosensitive pathways involved in myocardial cardiac fibrosis.

Gelatin-mTG hydrogels offer a versatile alternative [58,59] to materials such as PDMS, which are stiffer than native heart tissue, or gelatin methacryloyl (GelMA) hydrogels that typically require UV exposure for crosslinking. This work characterizes gelatin-mTG hydrogel chips fabricated through direct mix or immersion crosslinking methods. The impact of UVO exposure on the mechanical modulus of gelatin hydrogels was also characterized in this study. Further, this study presents two mechanical testing methods, Piuma and Instron, for characterizing gelatin hydrogels which can be compared against reported heart modulus values. We assert that the methods presented here can be used to develop cardiac tissue chip models and provide insight into the fabrication considerations that may impact material modulus.

## 5. Conclusions

This study focuses on characterizing gelatin-mTG hydrogels for use in cardiovascular disease models. We report the following: (1) Gelatin- mTG hydrogels exhibit a temporal stiffening, while 4% gelatin hydrogels were closer to the reported modulus of the native heart, these hydrogels were too fragile for chip fabrication processes, (2) the immersion crosslinking method resulted in higher modulus hydrogels, (3) incubation solutions impact the modulus of hydrogels, this may partially be attributed to modulations in pH, and (4) substrate modulus can impact SMA expression within cardiac fibroblasts. Future studies will incorporate vascular cells, cardiomyocytes, and fibroblasts into an engineered cardiac tissue model to investigate fibrosis.

## Figures and Tables

**Table 1:** Summary of Study.

[Gelatin] (*w*/*v*)	[mTG] (*w*/*v*)	Testing Parameters
2%	1%	Crosslinking method: Immersion vs. Direct mix
4%	0.8%	UVO exposure impact
5%	0.8%	Incubation solution: PBS vs. M199
10%	1%	Mechanical: Piuma vs. Instron

**Table 2:** Comparison of the Piuma and Instron mechanical testers.

	Pros	Cons
Instron	Measurement time duration Handles a wide range of materials and loads Versatile (compression, tension, flexural, cyclic testing)	Amount of material needed for measurements Can be destructive to samples Less sensitive for soft materials Larger instrument footprint
Piuma	Less sample required for measurements Possibility for higher resolution Lower cost alternative to AFM Measure in physiological conditions Automation for sample mapping Minimally Destructive Semi-portable	Optimized for air measurements Calibration time Cost of probes Probe to probe variability Sensitivity to vibrations and sample heterogeneity

## Data Availability

The original contributions presented in this study are included in the article. Further inquiries can be directed to the corresponding author.

## Abbreviations

The following abbreviations are used in this manuscript:

ECM	Extracellular Matrix
PDMS	Polydimethylsiloxane
mTG	Microbial transglutaminase
UVO	Ultraviolet Ozone
SMA	Smooth Muscle Actin
SD	Standard deviation
FDA	Food and Drug Administration
min	Minute(s)
h	Hours (s)
M199	Medium 199
PBS	Phosphate Buffered Saline
GelMA	Gelatin Methacryloyl
*w*/*w*	Weight/weight

## Appendix A

### Appendix A.1. Assessing mTG Incubation for Crosslinking

A 5% gelatin hydrogel was cast onto glass slides as described in Figure 1. The gelatin thin films were then incubated in a 0.8% mTG bath at room temperature for 10 min, 30 min, 1 h, 2 h, or 24 h to determine a suitable crosslinking time. Immediately after crosslinking, the gelatin thin films were rinsed with room temperature PBS and the modulus was measured before and after 3 min of UVO sterilization. Samples were incubated in PBS in cell culture conditions, and the modulus was measured every 24 h to assess thermal stability. Results suggest that a 2-h incubation time is sufficient for thermal stability and for attaining a physiologically relevant gelatin modulus.

**Table A1:** Average modulus for Gelatin- mTG hydrogels.

		Modulus based on Crosslinking Method	
[Gelatin] *w*/*v*	[mTG] *w*/*v*	Direct Mix	Immersion
2%	1%	2.2 ± 0.2 kPa (Piuma, PBS)	5.5 ± 0.5 kPa (Piuma, PBS)
		1.8 ± 0.3 kPa (Piuma, M199)	4.1 ± 0.6 kPa (Piuma, M199)
4%	0.8%	10.4 ± 1.0 kPa (Piuma, PBS)	13.0 ± 0.4 kPa (Piuma, PBS)
		8.4 ± 0.3 kPa (Piuma, M199)	7.5 ± 0.8 kPa (Piuma, M199)
5%	0.8%	13.7 ± 2.6 kPa (Piuma, PBS)	26.9 ± 8.6 kPa (Piuma, PBS)
		14.5 ± 0.8 kPa (Piuma, M199)	21.6 ± 5.1 kPa (Piuma, M199)
		15.1 ± 3.9 kPa (Instron, PBS)	
10%	1%	50.8 ± 12.1 kPa (Piuma, PBS)	52.2 ± 16.0 kPa (Piuma, PBS)
		47.8 ± 2.3 kPa (Piuma, M199)	39.2 ± 6.2 kPa (Piuma, M199)
		48.5 ± 10.9 kPa (Instron, PBS)	

### Appendix A.2. Enzymatic Degradation of Hydrogels

The immersion method was used to create 5% gelatin thin films. The thin films were incubated in M199 media with a 1 ng/mL collagenase solution to model enzymatic degradation of the hydrogel (Figure A5). By 72 h of incubation, the modulus of the 5% gelatin hydrogel significantly decreased from approximately 16 kPa to 9 kPa. The 10% hydrogel exhibited a significant decrease in modulus from 43 kPa to 19 kPa over the duration of the study. The Piuma nanoindenter can capture changes in surface modulus over time in this simulation of cell-induced enzymatic degradation.
